# Epidemiology of Abusive Head Trauma Among Children in Saudi Arabia

**DOI:** 10.7759/cureus.19014

**Published:** 2021-10-24

**Authors:** Fahad S Alanazi, Hassan Saleheen, Majid Al-eissa, Abdullah A Alshamrani, Ahmed A Alhuwaymani, Waleed K Jarwan, Mohammed S Hamaid

**Affiliations:** 1 College of Medicine, King Saud Bin Abdulaziz University for Health Sciences College of Medicine, Riyadh, SAU; 2 Public Health, King Abdullah International Medical Research Center, Riyadh, SAU; 3 National Family Safety Program, King Abdulaziz Medical City Riyadh, Riyadh, SAU; 4 Department of Pediatrics, King Abdullah Specialized Children’s Hospital, Riyadh, SAU

**Keywords:** victims, infant, saudi arabia, children, abusive head trauma

## Abstract

Background and objectives:To examine abusive head trauma (AHT) trends using data obtained from hospital-based child protection centers (CPCs) and the distribution of age in months among young children in Saudi Arabia.

Methods: A retrospective study was conducted that includes data obtained from 58 hospital-based CPCs in all 13 regions of Saudi Arabia registered in the National Family Safety Registry from 2010 to 2020. AHT cases (n=106) were identified for inclusion in the registry by a daily review of the emergency department logbooks.

Results: Over the 11-year period, there was a sharp increase in the number of cases, specifically after 2014, from seven cases per year in 2010 to 16 cases in 2020. AHT affects predominantly children aged 0-12 months (72.6%), followed by 13-24 months (17.9%), 25-36 months (3.8%), 37-48 months (3.8%), and 49-60 months (1.9%). Victims were characterized by a predominance of crying infants (23.6%), past history of abuse (13.2%), a child’s chronic disease and disability (7.6%), and prematurity (2.8%).

Conclusion: Different training and educational programs need to be performed to raise awareness of AHT. Enacting the pediatrician’s mandatory reporting law will improve the rate of reporting cases.

## Introduction

Abusive head trauma (AHT) is the deadliest form of child maltreatment (CM) and is the leading cause of death among the victims [1]. In the United States (US), according to the Centers for Disease Control and Prevention (CDC) [2], AHT can be defined as an injury to the skull or intracranial contents of children <5 years due to intentional abrupt impact and/or violent shaking. Recent epidemiological studies have identified mothers <18 years, mothers with low education, male caregivers, caregivers of substance abuse or mental health disorders, single-parent families, families with low socio-economic status, multiple gestations, and non-European American descent as the risk factors for AHT [3,4]. There are some characteristics among children that appear to increase the probability of AHT including age <1 year, male, and premature birth or low birth weight [4]. Previous research reported that AHT children - of all severities of injury - have 20% mortality and almost 50% permanent disability rates [5]. The consequences of AHT are severe including learning difficulties, hearing and physical disabilities, cerebral palsy, vision and speech problems, seizures, cognitive impairment, and death [6]. Baeesa and Jan reported characteristic signs such as altered level of consciousness, retinal/intracranial hemorrhages to those who were suspected to have AHT [7]. Furthermore, the average hospital charge for AHT patients was approximately $30,000 [8].

The incidence of admitted AHT children (per 100,000 children) was reported from 12.5 to 38.8 in many Western countries including the US (<1 year old) [3], United Kingdom (UK) (<2 years old) [9], and Australia (<2 years old) [10]. Nearly 80% of all AHT occurs among children age <2 years [11], with infants age <1 year having an incidence approximately eight times that of two years [3]. According to CDC, the incidence in the US was 0.76 fatal cases of AHT per 100,000 children <4 years, increasing to 2.14 when considering only children <1 year [12]. In Scandinavian countries, there is a lack of reports from population-based studies in terms of the incidence of AHT [13]. Myhre et al. reported a retrospective case series of infants and toddlers admitted with AHT to a tertiary intensive care unit in Norway [14]. In Sweden, Tingberg et al. conducted a retrospective medical record review of infants presenting to a pediatric emergency department with a head injury and revealed that 54% identified infants had a history that should have prompted suspicion of AHT [15]. Another study in Estonia reported an incidence of AHT (28.7 per 100,000) among children ≤1 year [16].

It is obvious from the literature review that there is a lack of publications on AHT that have emerged from Middle Eastern countries where children and adolescents comprise a large proportion of the population [17]. A regional study conducted in Bahrain reported that most of the children (78%) were ≤1 year old and the mortality rate was 17% [1]. In Saudi Arabia (SA), AHT was first recognized in 1994 [18]. National hospital-based registry reported that AHT represents 5% of the physical abuse. The suspected perpetrators include males (60%), unemployed, and/or with low levels of education of parents/caregivers. The mortality rate was 25% and most of the surviving children (70%) were discharged with moderate to severe neurological disorders [13].

While there are numerous publications of a clinical nature on AHT, few population-based epidemiological studies exist that enable to estimate the frequency and identifying its risk factors. Identifying the issue and its characteristics is important to increase awareness and recognition. However, there is little knowledge of such a context in SA making it an area in urgent need of exploration. In this study, we report the demographic characteristics, incidence, risk, and outcome for AHT cases in SA.

## Materials and methods

A retrospective study was conducted that includes data obtained from 58 hospital-based Child Protection Centers (CPCs) in all 13 regions of SA registered in the National Family Safety Registry (NFSR) from 2010 to 2020. The NFSR is a web-based online disease registration system that collects data from CPCs and Adult Protection Centers within the SA. AHT cases (<5 years) were identified for inclusion in the registry by a daily review of the emergency department logbooks by the suspected child abuse and neglect (SCAN) team - a multidisciplinary team that includes physicians, nurses, and social workers with extensive experience in the management of CM cases.

Data collected include demographic characteristics of the children (age and gender), information of caregivers and perpetrators, history of previous abuse, and fatality. Descriptive analysis was conducted for the study variables.

All data entry forms have validation checks and warning messages that restrict users from making any data entry mistakes. Validation rules are designed as a quality check of data entered in the database. The diagnosis validation rules that are integrated are run routinely to confirm accuracy. Since the registry collects personally identifiable health data, one of the major responsibilities of the common user is to ensure attention to privacy as a fundamental consideration in the collection and maintenance of the information obtained. The NFSR ensures that only authorized individuals should handle the raw data and information managed by the registry database, and is accessible to the appropriate people through assigned passwords. After approval from the NFSR board, registry data are released to the researcher to ensure that the privacy of individuals does not supersede other rights or societal goals while carrying out the research.

The study obtained ethical clearance from the Institutional Review Board (IRB) of the King Abdullah International Medical Research Center (KAIMRC) in Saudi Arabia. Hospital medical record numbers were used without any identification material. The data were collected only for research purposes and were not used for other purposes. A series of measures were taken to ensure the protection of confidentiality. The data did not contain the name of the patient but were labeled with a reconstructable personal alphanumeric identifier. All data were stored on a designated computer with an access-limited locked hard drive.

The study was approved by the Institutional Review Board (IRB) of the King Abdullah International Medical Research Center (KAIMRC) at the Ministry of National Guard Health Affairs (MNGHA) in SA (Research protocol number - RC15/067).

## Results

The socio-demographic characteristics of the AHT victims (n=106) are shown in Table 1.

**Table 1:** Demographic information on abusive head trauma victims (n=106)

	Number (%)
Age (mean)	
Mean	6.1 (month)
Gender	
Male	56 (52.8)
Female	50 (47.2)
Living arrangement	
Both parents	90 (84.9)
Single parent	14 (13.1)
Other relatives	2 (2.0)
Parent’s highest level of education	
Primary school or less	17 (15.9)
High school	62 (58.9)
College or higher	27 (25.2)
Alleged perpetrator known	
Yes	56 (52.8)
Unknown	40 (37.7)
Perpetrator’s relationship to the child	
Biological father	20 (35.8)
Biological mother	7 (12.5)
Stepfather	8 (14.3)
Stepmother	7 (12.5)
Sibling	4 (7.1)
Other caregivers (e.g., babysitter)	10 (17.8)
Health outcome	
Well	78 (73.6)
Disability	17 (16.0)
Death	11 (10.4)

The mean age of the victims was 6.1 months and 52.8% were male. More than three-quarters (84.9%) of participants lived with both parents, 13.1% with a single parent, and 2% with other relatives. Regarding parents’ highest level of education, over half (58.9%) had completed high school education, followed by the college or higher degree (25.2%), and primary school or less (15.9%). Among children who had experienced AHT, biological father was the perpetrator in 35.8% of the cases, followed by biological mother (12.5%), step-father (14.3%), and step-mother (12.5%). In terms of health outcomes, over a quarter (26.4%) of the victims were either disabled or died.

**Figure 1:**
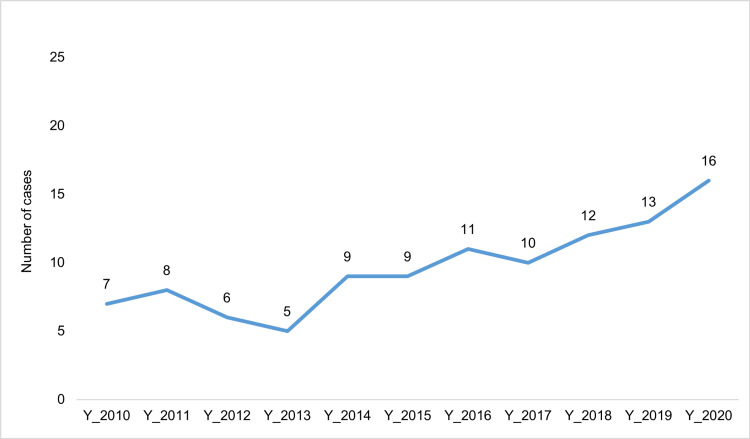
Estimated number of cases of abusive head trauma (2010-2020)

Over the 11-year period, there was a sharp increase in the number of AHT cases, specifically after 2014, from seven cases per year in 2010 to 16 cases in 2020 (Figure 1).

**Figure 2:**
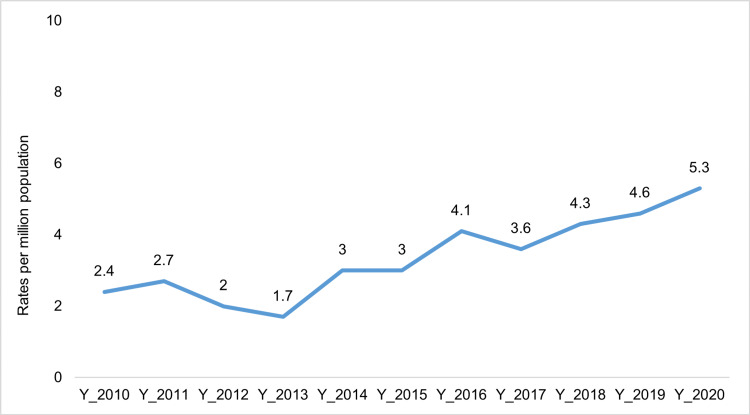
Estimated rates of abusive head trauma (2010-2020)

The year-to-year variation in AHT rates is shown in Figure 2.

**Figure 3:**
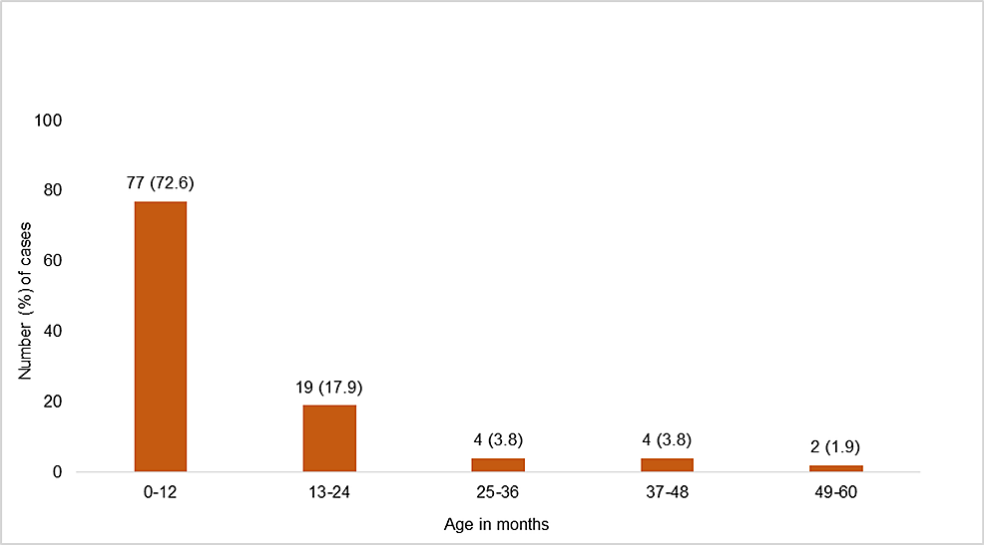
Age distribution in months for abusive head trauma cases (n=106)

AHT rates were calculated from child population data ranging from 2.4 to 5.3 per million population between 2010 and 2020 which indicate that AHT rates varied widely from year to year. Figure 3 shows the age distribution in months for AHT cases across the study population.

AHT affects predominantly children aged 0-12 months (72.6%), followed by 13-24 months (17.9%), 25-36 months (3.8%), 37-48 months (3.8%), and 49-60 months (1.9%). Victims of AHT were characterized by a predominance of crying infants (23.6%), past history of abuse (13.2%), a child’s chronic disease and disability (7.6%), and prematurity (2.8%). Among the 106 cases, the suspected perpetrators of AHT incidents were the young aged parents (13.2%; Table 2). 

**Table 2:** Characteristics of the children and their families (n=106)

	Number (%)
Crying infant	25 (23.6)
Past history of abuse	14 (13.2)
Parent’s young age	14 (13.2)
Child’s chronic disease or disability	8 (7.6)
Prematurity	3 (2.8)
Others	42 (39.6)

## Discussion

AHT results in severe injuries to the skull and intracranial contents and represents a significant disease burden in infants and children. To the best of our knowledge, this hospital-based child protection center retrospective study is the first published report in SA among infants and children which reports rates of AHT over the last decade and characteristics of the children and their families. Our study results have three major findings. First, a large proportion of AHT victims identified were infants and males. Second, an increase in the number of AHT cases was found from year to year. Third, crying infants were the main victims of AHT.

AHT is largely restricted to infants and males. Children aged <1 year are more likely to suffer brain injuries usually caused by CM than accidents [19]. Results from our study are consistent with those of Parks et al. who reported most children who have fatal AHT aged <12 months and are males [12]. In Estonia, Talvic et al. found that all of the 26 AHT cases that were discovered and diagnosed between 1997 and 2003 were <1 year [16]. A study conducted in Turkey stated that 88% of all AHT cases were under the age of two years and males represented 56% of the cases [20]. According to Frasier [21], the increased vulnerability may be related to infants’ close contact with their caregivers and need constant care as compared with older children. In addition, infants’ heads are comparatively larger and brain is proportionately heavier for their body. Their neck muscles are not strengthened, since the ability to sustain the head upright develops from 2 to 4 months [22]. Prevention strategies for AHT involve educational actions for parents of newborns before discharge from the hospital which include videos and pamphlets disclosing the risks of AHT, the pattern of crying, and the strategies that parents can utilize when they feel frustrated with the crying baby might reduce the incidence of AHT among male infants [23]. Dias et al. also revealed a decrease in AHT using written and video content about the consequences of shaking in addition to asking parents to voluntarily sign a commitment statement acknowledging receipt and understanding of the information [24].

Consistent with Kelly et al. report [25], we found an increase in the incidence of AHT over time. In another study, AHT rates from hospital discharge data ranged from 25.0 to 44.9 per 100,000 person-years between 2000 and 2011 [26]. These rates might be underestimated as many cases are not recognized as having been caused by CM or do not come to the attention of health care providers to be identified [12,21]. Pediatricians should conduct a thorough and objective medical evaluation of the babies with clinical features of potential AHT. They should consider consulting a physician specialized in CM to ensure the accuracy of the diagnosis. They should understand that legal burdens of proof are not required for the diagnosis. They are required to report suspected CM to child protective services if a final diagnosis of CM has been made or not. As mandated reporters, they carry the burden of recognizing and responding to medical manifestations of AHT [27].

Crying is an important mode of communication for babies that plays an important role to ensure survival, health, and development when they depend on their caregivers [23]. Although crying is normal for the initial period of their life, previous studies have reported that specific crying properties can cause frustration and stress for the caregivers, the stimulus that contributes to the occurrence of AHT [28]. Adamsbaum et al. have indicated that crying was the reported stimulus in 63% of the cases that was comparable to Flaherty’s study where in 67% of confessions, crying was the circumstance that triggered the maltreatment [29,30]. However, our study reported that only 23.6% of crying infants were the main victim of AHT. It is anticipated that lack of consistency in the prevalence rate was due to knowledge and perception of different crying properties that contribute to the occurrence of AHT. There is an urgent need for a meta-analysis of all available articles to explore the phenomenon among infants. The prevention programs need to combine the understanding of specific properties of crying during the initial period of a baby’s life, the occurrence of inconsolable crying during their development, and the negative impact of crying during this period is the occurrence of AHT and other types of CM [28]. A universal AHT prevention program, the Period of PURPLE Crying, the objectives of which are to support caregivers in their understanding of early increased crying in infancy and to reduce the incidence of AHT [26]. According to Dias et al. [24], it has been hypothesized that this intervention would reduce AHT by 50% from a previously determined baseline. This intervention program can be recommended for caregivers in SA and should be customized and implemented.

The study has some limitations. First, this is a retrospective review of a registry database that does not capture comprehensive information on the AHT. Second, the AHT cases are the selected sample from the emergency department. The sample does not include those who died of AHT before they were admitted to the hospital. Therefore, the results may not represent the number of infants/children in the community. Third, the retrospective data obtained were limited to the quality of the documentation in the medical record. Fourth, this study cannot establish a causal relationship.

## Conclusions

The results of this study indicate that AHT cases increase over time in SA and need more attention of the health care providers, child protection workers, counselors, law enforcement personnel, and policy makers. Prevention and detection of AHT should be priority in terms of future management. Preventive measures need to focus on caregivers of the babies. Different training and educational programs need to be performed to raise awareness of AHT. Enacting the pediatrician’s mandatory reporting law will improve the rate of reporting AHT cases. Future studies are required to address the effect of AHT in the context of local culture.
